# Biopolymer- and Natural Fiber-Based Biomimetic Tissues to Realize Smart Cosmeceuticals and Nutraceuticals Using an Innovative Approach

**DOI:** 10.3390/pharmaceutics15112525

**Published:** 2023-10-24

**Authors:** Pierfrancesco Morganti, Maria-Beatrice Coltelli, Alessandro Gagliardini, Andrea Lazzeri, Gianluca Morganti, Giovanna Simonetti, Tilman Fritsch, Vittorio Calabrese, Alessandra Fusco, Giovanna Donnarumma

**Affiliations:** 1 R&D Unit, Academy of History of Healthcare Art, 00193 Rome, Italy; morgantipf@gmail.com; 2 Dermatology Department, China Medical University, Shenyang 110122, China; 3 Department of Civil and Industrial Engineering, University of Pisa, 56122 Pisa, Italy; andrea.lazzeri@unipi.it; 4 R&D Unit, Texol Srl, 65020 Pescara, Italy; alessandro.gagliardini@texol.it; 5 ISCD Nanoscience Centre, 00165 Rome, Italy; lucamorgan@libero.it; 6 Environmental Department Biology, La Sapienza University, 00185 Rome, Italy; giovanna.simonetti@uniroma1.it; 7 NAM Institute, A-5020 Salzburg, Austria; tilmanf@hotmail.com; 8 Department Biomedical and Biotechnological Science, School of Medicine, Catania University, 95123 Catania, Italy; vittorio.calabrese@unict.it; 9 Department of Experimental Medicine, Campania University Luigi Vanvitelli, 80138 Naples, Italy; alessandra.fusco@unicampania.it (A.F.); giovanna.donnarumma@unicampania.it (G.D.)

**Keywords:** pullulan, bamboo, electrospinning, fish collagen polypeptide, allantoin, niacinamide, anti-inflammatory, antioxidant, cosmeceutical

## Abstract

More sustainable and smart cosmeceuticals and nutraceuticals are necessary due to the ecological transition. In this study, a pullulan-based water solution containing chitin nanofibril–nano-lignin (CN-LG) complexes that encapsulate fish collagen polypeptide, allantoin and nicotinamide was electrospun onto a nonwoven substrate made of bamboo fibers to obtain a smart nanostructured bilayer system for releasing active molecules onto the skin or other body tissues. Infrared spectroscopy was used to characterize the composition of the bilayer system before and after rapid washing of the sample with distilled water and liquids mimicking physiological fluids. The viability of keratinocytes was studied as well as the antioxidant activity, protective activity towards UV light, metalloproteinase release of aged fibroblasts and the inhibitor activity against collagen degradation. Immunomodulatory tests were performed to investigate the anti-inflammatory activity of the bilayer system as well as its indirect antimicrobial activity. The results indicate that the bilayer system can be used in the production of innovative sustainable cosmeceuticals. In general, the adopted strategy can be extended to several smart treatments for fast release that can be commercialized as solid products, thus avoiding the use of preservatives and water.

## 1. Introduction

The so-called green revolution [1] underlines the urgent need to find new approaches for using innovative and natural carriers of active ingredients that are indispensable to increase and ameliorate the production of food and other industrial goods including cosmetics and diet supplements [2]. New approaches have to be realized without diminishing the Earth’s natural materials, but rather by producing goods using waste, according to the principles of a circular economy [3]. Different from today’s linear economy, which is based on take, make and producing waste, a circular economy is based on redesigning, remaking, reusing and recycling [3,4]. It has been estimated, in fact, that because of today’s approach of producing and consuming, approximately a third of the world’s food is lost every year, and 14% is lost before reaching the retail level [5]. Thus, 1.3 billion tons of waste are generated yearly, the majority of which remains in the land as dangerous pollution or represents a burden for waste management [4,5]. Fossil-based plastics present in many industrial products represent another important issue for the environment [6]. In general, as a consequence of diffused pollution, fish and marine mammals are continually eating microplastics as food. In addition, microplastics [7] have also been recovered in tea [8] and at the level of the human placenta [9]. Fortunately, progress in nanotechnology over the last twenty years has resulted in the discovery, engineering and production of innovative delivery systems such as biodegradable, smart and specialized nonwoven tissues and films [10,11,12,13]. Such delivery systems can load and carry active ingredients, and then release them at the right site, at the right concentration, for a sufficient correct period of time, according to the so-called 4Rs delivery (right chemical, right site, right concentration and correct period of time) [14]. Skin adhesion and penetration, in fact, is conditioned by the physicochemical properties of the selected ingredients, such as their polarity, shape, size and zeta potential together with the carrier system vehiculating molecule(s) on the skin at the dosing condition [15,16]. However, it is interesting to underline the capacity of some collagen peptides (CP) to enhance the diffusivity through the skin layers of various active ingredients, with an efficiency that depends on the manufacturing procedures and the selected ingredients. The penetration ability of CP seems to be attributable to their amino acid sequence, mainly the one of the essential amino acids [17]. The presence of positively charged arginine groups, in fact, might enhance their ability to bind to negatively charged cell surfaces by electrostatic interactions further helped by chitin nanofibrils (CN) as a component of the carrier [16,17]. However, further studies are necessary to understand the exact mechanism of the peptides and the other active ingredients that mediate transport from a tissue to non-viable and viable layers of the skin. Nevertheless, there is a need to discover new active ingredients and natural-oriented carriers according to the new green way of living.

Recently, the study of buccal films as an alternative medication delivery technique has increased for several medications, including anti-inflammatory, anesthetic, and protein and peptide therapies [18]. These oral thin films are similar to dissolving systems because they are drenched in saliva and adhere to the application site. The release speed must be fast (so that direct swallowing is limited), and afterwards, the active molecules are administered by oromucosal absorption [19]. Innovative smart delivery systems could also be useful for this application; both the polymers used as carriers and the active ingredients loaded on should be correctly selected.

The electrospinning process has been used for producing smart delivery systems because it can produce smart, nanostructured and sustainable tissue carriers [20,21,22]. These innovative vehicles that are produced using micro-nanofibers can embed chitin nanofibril–nano-lignin (CN-LG) complexed particles, which can encapsulate selected active ingredients [23,24]. The productivity of electrospun tissue has enormously increased in the last decade but it remains to be a critical point for industrial applications [25]. Therefore, application of a thin electrospun functional layer onto a suitable nonwoven substrate can result in an innovative tissue for smart release with better scalability. Bamboo nonwoven textiles are very well known because of their interesting properties [26]. Bamboo fibers have various and numerous micro gaps. With respect to cotton, bamboo increases its moisture absorption and makes it softer. Bamboo fiber is highly durable, stable and tough, and it has high tensile strength. It is also sustainable and biodegradable. Moreover, bamboo fiber tissues are antifungal, antibacterial, bacteriostatic, hypoallergenic, hydroscopic, a natural deodorizer and resistant against ultraviolet light [27]. Due to its antibacterial feature, it is used for making masks, bandages and sanitary napkins. The tissue, after the application of the bilayer system, can be recycled or composted thanks to the durability and biodegradability of the bamboo fibers, respectively.

Specifically, one recent innovative tissue was obtained by electrospinning water-soluble pullulan (PUL) and successively inserting CN-LG complex powder into the PUL tissue using dry powder impregnation, a methodology based on the application of an alternating electric field [28]. This product, prepared in two steps, showed the advantage of being solid; therefore, it did not require any preservative (often causing allergies and dermatitis) or gas barrier plastic-based packaging, and it could be used on the wet skin forming an emulsion that could be removed after the application. The aim of the present study is to design and realize, in only one step, an innovative biodegradable pre-aging cosmeceutical product that is effective for photo-aged and/or prematurely aged skin, which presents an altered synthesis of collagen and protective antioxidant enzymes and molecules, according to recent trends regarding anti-aging, pro-aging and pre-aging considerations [29,30]. Thus, to make this tissue, pullulan (PUL), as a basic polymer, was used because of its skin-adhering facilitation and UV-light barrier properties, as well as its antioxidant and antibacterial activities [31]. The idea was that PUL could embed the CN-LG complex, known for its antioxidant and skin repairing activities, enhanced by encapsulated active ingredients such as nicotinamide, collagen polypeptides and allantoin [12,13,32]. Nicotinamide, as a precursor of nicotinamide adenine dinucleotide (NAD), is an essential cofactor for ATP synthesis, downregulated in photoaged subjects. Moreover, its capacity to boost the cellular NAD concentration facilitates DNA damage repair of aged and photo-aged skin, inducing its synthesis and reducing the UV immunosuppression activity [33,34,35]. In addition, fish collagen peptides have been shown to prevent skin-aging effects, influence collagen turnover/balance, increase skin elasticity and its antioxidant activity and inhibit tyrosinase activity, thus, reducing wrinkle formation [36,37,38]. Moreover, allantoin can stimulate fibroblast proliferation, which might increase elastin and collagen synthesis, thus enhancing cell growth [39]. Finally, it is interesting to underline how the combined activities of collagen peptides and allantoin seem to increase skin penetration of the active ingredients [40,41]. In addition to producing the bilayer, the present study aimed to characterize the bilayer system. Thus, after assessing the capability of the new bilayer system to release active molecules in body fluids (so that it can be exploited in several smart release cosmeceutical or nutraceutical applications), the first parameters investigated in vitro were the antioxidant activities of both the selected block polymeric nanoparticles and the final realized tissue together with their safeness and effectiveness. Moreover, the enzymatic activities of metalloproteinase and collagenase were studied to verify the eventual effectiveness of the product for regulating the imbalance between proinflammatory and anti-inflammatory cytokines and the collagen synthesis, always present in photo-aged and aged skin (or mucosa) [40].

## 2. Materials and Methods

### 2.1. Materials

Pullulan was obtained from Shandong Freda Biotechnology Co., Ltd, Linshu, China; fish collagen polypeptides were obtained from Rousselot Angouleme S.A.S., Angouleme, France; nicotinamide was obtained from Sigma-Aldrich, Milano, Italy; allantoin was obtained from Akema Srl, Coriano (RN), Italy; CN-LG complexes were obtained from the ISCD Nanoscience Center, Rome, Italy; Dulbecco’s phosphate buffer (pH 7.2–7.8) was purchased from Sigma-Aldrich.

### 2.2. Methods

**Figure 1:**
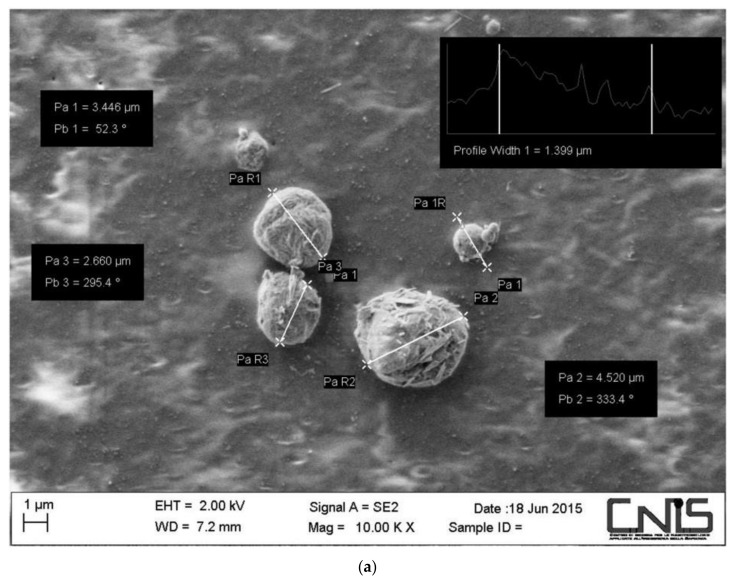

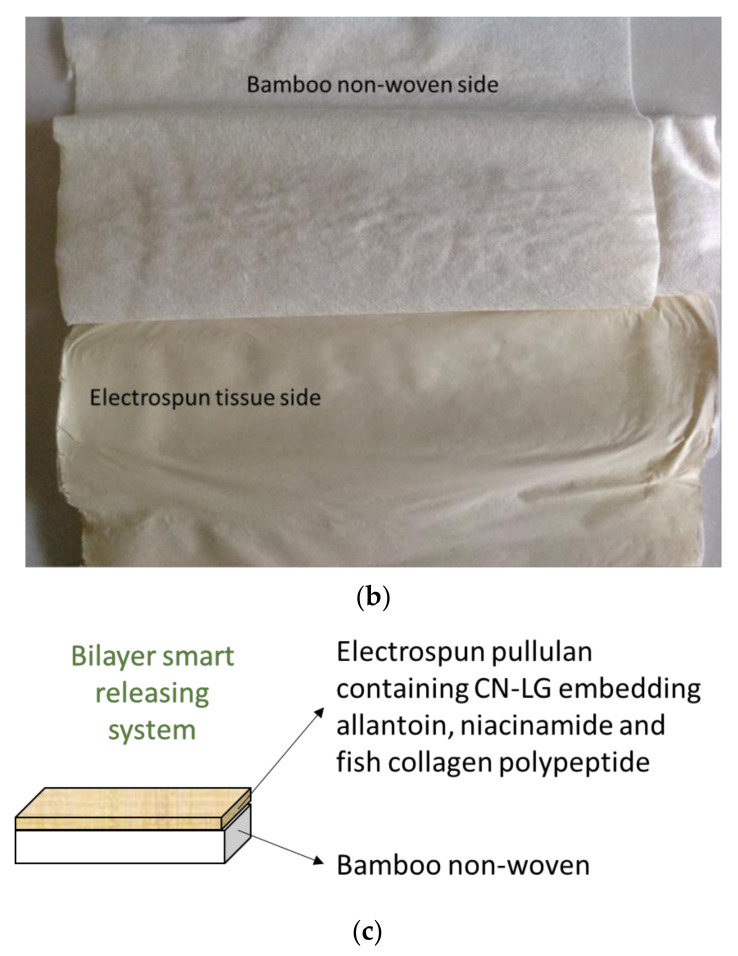
(**a**) SEM micrograph related to CN-LG complexes; (**b**) picture of the bamboo nonwoven substrate modified on its surface by electrospinning of a pullulan tissue layer containing CN-LG complexes and active molecules; (**c**) scheme of the developed bilayer system.

The synthesis of nanostructured particles, based on CN-LG complexes encapsulating the active ingredients, was performed using the slow-stirring method to add the alkaline solution of negatively charged lignin (2% *v*/*v*) into a stabilized acidic suspension of positively charged CN (2% *v*/*v*) (containing nicotinamide and allantoin previously solubilized). The solution was dropped using a 30-gauge needle under high speed and constant stirring, at temperatures of 50–60 °C for 1 h, according to our previous studies [10,32]. The obtained nanostructured micrometric particles, purified by centrifugation, re-suspended in distilled water and spray dried, were characterized by scanning electron microscopy (SEM) (Philips XL20, Amsterdam, The Netherlands), as reported in Figure 1a. The particles’ mean sizes were measured using a Zetasizer (NanoZS Model 3600-Malvern Instruments, Worcestershire, UK) and their release was measured using a dissolution apparatus (Distek 2100 B) and controlled by high-performance liquid chromatography (HPLC) (Varian 9012, Varian Associated Inc., Palo Alto, CA, USA). The loading capacities of the entrapped active ingredients were determined by gel filtration chromatography and analyzed using a spectrophotometer, according to our previous studies [32]. The results are reported in Table 1. The particles obtained by the gelation method and mixed with the gel solution of pullulan were electrospun using an Elmarco NS 1S500U at a speed of 50 mm/min onto a nonwoven substrate made of bamboo fibers to obtain the samples reported in Figure 1b. The grammage of the electrospun tissue was measured by weighting 5 specimens of known dimensions, and the result was 0.9 × 10^−3^ g/cm^2^. The grammage of the bamboo nonwoven substrate, kindly provided by ORMA srl (Matera, Italy), was 30.5 g/m^2^.

The functional tissue was characterized by infrared spectroscopy using a Nicolet T380 Thermo Scientific instrument equipped with a Smart ITX ATR accessory with a diamond plate (Thermo Fisher Scientific, Waltham, MA, USA), collecting 256 scans at 2 cm^−1^ resolutions. A small amount of tissue was put in contact with the diamond plate to analyze the sample surface. Spectra were elaborated by using the EZ OMNIC 32 software.

Qualitative release tests supported by ATR-IR characterization were carried out. Saliva is a colorless, slightly stringy liquid with a reaction close to neutral (pH 6.6 on average), made up of 98.7% water, 0.8% inorganic substances (chlorides, carbonates, bicarbonates, phosphates, etc.) and 0.5% from organic substances (mucin, enzymes, especially ptyalin, albumin, etc.). To mimic saliva, a solution of distilled water containing 0.8% sodium chloride was considered and 0.1 mL of a water solution of HCl 0.0025 M was added to 1 L of solution to reach a pH of 6.6. A solution of 0.9% NaCl and distilled water was also used for comparison. One strip of the electrospun brown tissue deposited onto white bamboo tissue (10 mm × 30 mm) was rinsed in 10 mL of liquid. After 10 s, the strip was removed from the liquid and the color was analyzed. The test was repeated two times for the three liquids and also for the distilled water.

The antioxidant activities of the CN-LG nanoparticles, CN-LG-entrapping polypeptides, nicotinamide, allantoin and the PUL tissue embedded with nanoparticles were evaluated, in vitro, using the total antioxidant potential (TRAP) technique modified by our group, as previously reported [32]. The principle of the method is based on the peroxidation of linoleic acid by the oxidant compound 2,2-azobis-2,4-dimethylvaleronitrile (AMVN) with the formation of malondialdehyde (MDA) as the final product. Then, 10 mM of linoleic acid was dissolved in 1 mL of methanol, dried under nitrogen and redissolved in 2 mL of phosphate buffer. Lipid peroxidation was induced by adding 10 mM of AMVN for 15 min at 37 °C to samples containing 10 ng of CN-LG and CN-LG enriched with the active ingredients and the final tissue, respectively. The control consisted of linoleic acid peroxidated with 10 mM of AMVN for 15 min at 37 °C. The formation of MDA was detected by following the Ursini fluorometric method [42].

### 2.3. Cell Culture

According to our previous studies, the HaCaT keratinocyte cell line was grown in 9BM medium supplemented with 10% (*v*/*v*) fetal bovine serum at 37 °C and 5% CO_2_ [43]. Then, 10 mg/mL of nanoparticles and tissue were added to the cell culture and incubated for 48 h in a 37 °C humidified atmosphere containing 5% CO_2_. Viability was assessed by using the modified MTT method. Tests were carried out in triplicate.

Additionally, after the nanostructured tissues were sterilized under UV light, the immortalized human keratinocyte HaCat cell line was cultured in Dulbecco’s modified Eagle medium (DMEM) supplemented with 1% PenStrep, 1% glutamine and 10% fetal calf serum (Invitrogen, Carlsbad, CA, USA) at 37 °C in air and 5% CO_2_.

#### 2.3.1. Alamar Blue Assay

The HaCaT cells, cultured as described above and seeded in 12-well plates until 80% confluence, were incubated for 24 h with the fibers. At the end of this time, resazurine was added to a concentration of 0.5 mg/mL and incubated for 4 h. The Alamar Blue test is based on a redox indicator that changes color according to cell metabolic activity. The supernatants were read with a spectrophotometer using a double wavelength reading at 570 nm and 600 nm. Finally, the reduced percentage of the dye was calculated [43] by correlating the absorbance values and the molar extinction coefficients of the dye at the selected wavelength, following the protocol provided by the manufacturer. The results obtained are expressed as the percentage of Alamar Blue reduction (% AB_RED_), which is related to metabolically active cells. A value close to 100% indicated that the cell metabolic activity was not affected by the samples.

#### 2.3.2. UV-Irradiated Keratinocytes and Intracellular ATP Level Determination

According to Surjana et al. [34], the HaCaT keratinocyte culture, grown in Dulbecco’s modified Eagle medium supplemented with 10% (*v*/*v*) fetal bovine serum (FBS), was irradiated for 24 h at 37 °C, with a 4 J/cm^2^ source, using a 1000 W Xenon arc solar simulator, which matched the spectrum of natural sunlight. The concentration of FBS was reduced to 0.5% (*v*/*v*) 24 h before irradiation, in order to minimize the number of cells replicating DNA synthesis. Prior to irradiation for 24 h at 37 °C, samples were prepared by adding, respectively, 10 ng/mL of CN-LG carrier, CN-LG particles or the final tissue. The time between harvesting and irradiation was selected because it was necessary to obtain a maximum rate of relative ATP synthesis, and lesion repair by keratinocytes was detected using comet assays and immunohistochemistry. Fewer photoproducts, including cyclobutene, pyrimidine dimers and 8-oxoguanosine were recovered compared to the untreated groups. These tests were carried out in triplicate.

#### 2.3.3. Metalloproteinase (MMPI) Recovery

According to our previous studies [32], the human fibroblast culture, after 24 h of incubation, was incubated for a further 2 h after the addition of 600 nM of H_2_O_2_, to obtain aged cells. Soon after, the medium was removed and replaced by fresh medium. After maintaining the culture for a further 144 h and substituting the medium after 70 h, both aged fibroblasts and untreated fibroblasts were detached with trypsin, seeded in 96-well microplates and cultured for 24 h. Soon after, the culture medium was replaced with DMEM medium, containing either transforming growth factor (TGF-beta) or PUL tissue, CN-LG carrier, CN-LG particles and the produced final tissue at a dose of 10 nM/mL, incubated for 72 h and compared to the untreated control. The MMPI release was tested in triplicate using an ELISA kit.

#### 2.3.4. Collagen Synthesis

According to our previous study, the collagen synthesis was controlled indirectly on the fibroblast cultures by measuring the hydroxyproline produced by the collagenase activity [29] via the modified Edwards and O’Brien method [44]. Using this method, fibroblast cultures were incubated with collagenase enzyme, alone and with CN-LG carrier, nanoparticles and the final tissue added in the quantity of 10 ng/mL. After hydrolysis and oxygenation, the red color obtained through the liberated hydroxyproline and the Erlich’s solution was quantified using a spectrophotometer at 560 nm, to verify the percent of collagenase inhibition. Higher collagenase inhibition and higher collagen synthesis activities were measured by the ELISA kit.

#### 2.3.5. Statistical Analysis

Data collected in Section 2.3.2, Section 2.3.3 and Section 2.3.4 and data about antioxidant properties were plotted using the GraphPad software Prism 9.5.1 (GraphPad Software Inc., San Diego, CA, USA) based on one-way or two-way ANOVA; the required corrections were applied using Turkey’s test, Bonferroni correction, Dunnet post-test and Newman–Keuls Q test.

### 2.4. Immunomodulatory Tests

To evaluate the inflammatory response and the production of HBD-2, the HaCat cells, plated in a 6-well plate at 80% confluence, were treated with fibers and sterilized, as previously described, for 6 and 24 h.

At the end of the experiment, the mRNA was extracted from the cells and the expression levels of the proinflammatory cytokines (IL-8, IL-6, IL-1α and TNF-α), anti-inflammatory cytokine TGF-β and antimicrobial peptide HBD-2 were evaluated by real-time PCR.

## 3. Results

Fish collagen polypeptide, nicotinamide and allantoin active molecules were entrapped in the CN-LG complexes as a result of the adopted gelation method which consisted of combining positively charged chitin nanofibrils in a slightly acidic solution (where active molecules were also suspended) and negatively charged nano-lignin. After homogenization, filtration, water washing and spray drying, nanostructured particles were obtained (Table 1).

The nanoparticles were characterized by SEM, and the size of the nanoparticles was determined to be 185 nm (Figure 1a). The yield of the produced nanoparticles was about 48%, probably because the precipitation of nanostructured complexes was partially hindered by the presence of active molecules. Nevertheless, the loading of complexes with polypeptide, nicotinamide and allantoin was effective and 65% of the active molecules were entrapped in the CN-LG complexes. The obtained nanostructured particles were suspended in a water solution containing pullulan, and the solution was electrospun to obtain a tissue. Due to the very low thickness and consequent fragility of the tissue and to develop a process with a high productivity, the tissue was electrospun onto a fully bio-based bamboo nonwoven substrate to obtain a fully bio-based bilayer system. For sublingual release of an ingredient, a bamboo non-soluble substrate can limit ingestion of active molecules.

**Figure 2:**
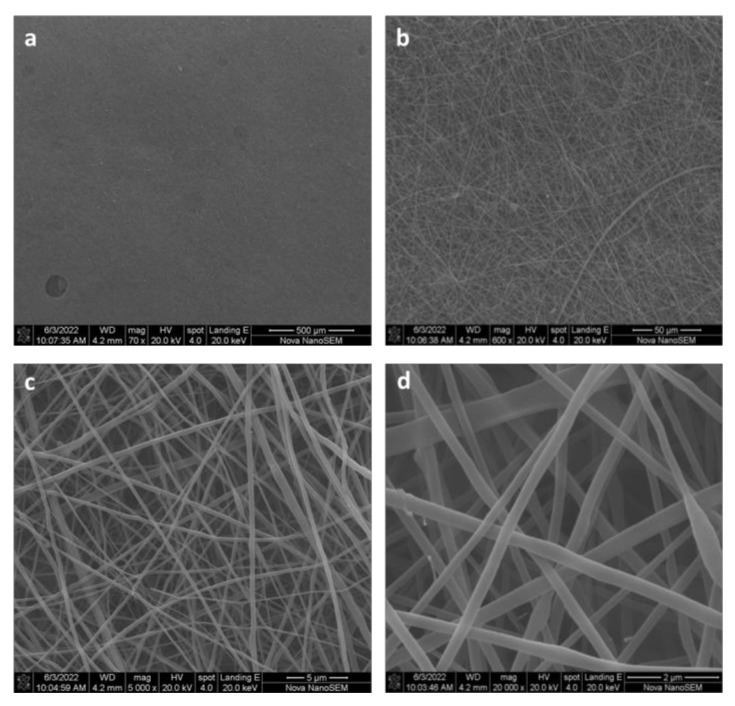
SEM micrographs showing the morphology of the electrospun pullulan tissue: (**a**) Magnification 70×; (**b**) magnification 600×; (**c**) magnification 5000×; (**d**) magnification 20,000×.

The electrospun tissue was characterized by SEM (Figure 2) and it was possible to observe the high regularity and submicrometric thickness of the electrospun filaments consisting of pullulan and containing CN-LG and active molecules. Teno et al. [19] electrospun pure pullulan to produce a nanostructured tissue, and then modified the tissue using dry powder impregnation. Electrospinning of the pullulan solution including suspended additives was difficult because of the scarce resistance of the filaments during the electrospinning process. In the present case, optimized conditions for the preparation of the sample were identified; therefore, active molecules were present in the pullulan fibers so that they could be released on wet skin or different body tissues, where the humidity of body fluids could dissolve the hydro soluble pullulan. The loaded NC-LG particles could not be observed in the nanostructured tissue because they were embedded in the sub-micrometric PUL filaments.

**Figure 3:**
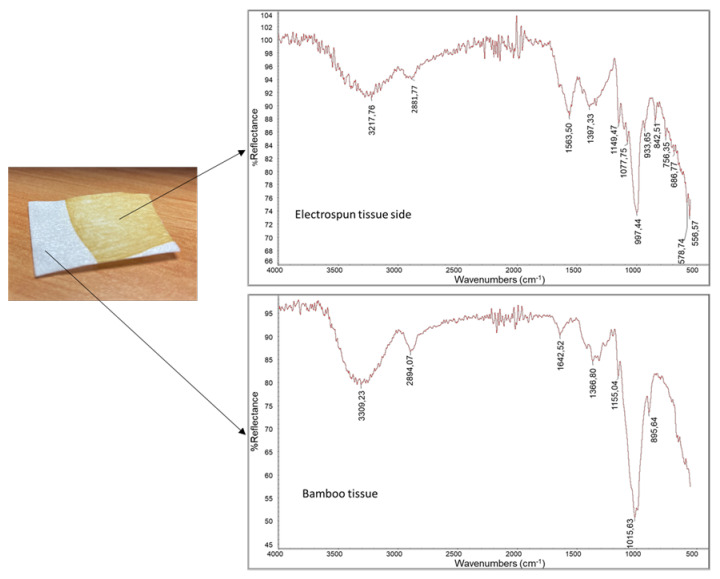
Infrared spectra recorded by ATR accessory for the brown pullulan-based layer and the white bamboo nonwoven substrate.

The sample was characterized by infrared spectroscopy using an ATR accessory to study the surface composition (Figure 3). The infrared spectra were recorded for the pullulan electrospun tissue and the bamboo nonwoven substrate. In the spectrum of the bamboo nonwoven substrate, the band at 3309 cm^−1^ can be attributed to the stretching of -OH groups present in cellulose, hemicellulose, lignin, phenolic compounds and carboxylic acid. The band at 2894 cm^−1^ is related to the C–H stretching. The peaks at 1642 and 896 cm^−1^ can be attributed to the O–H stretching vibration of absorbed water in carbohydrate and the C–H deformation vibrations of cellulose [45].

The bands at 1424, 1364 and 1157 cm^−1^ represent C-H deformation (methoxyl group in lignin), C-H deformation (symmetric) and C-O stretching of ester groups, respectively [46].

The analysis performed on the brown surface, where the electrospun layer was deposited, showed a different spectrum. The bands at 3309 cm^−1^ and 2882 cm^−1^ can be attributed to -OH stretching and C-H stretching in the pullulan-based layer, respectively. The 997 cm^−1^, 1077 cm^−1^ and 1147 cm^−1^ bands can be attributed to C-O and C-C bonds and deformational vibrations of the C-C-H, C-O-H and H-C-O bonds in the pullulan spectrum, as reported by several authors [47,48,49]. Additional broad bands at 1649, 1562 and 1397 cm^−1^ can be attributed to the CN-LG complexes and the presence of polypeptide, allantoin and niacinamide. Niacinamide, chitin nanofibrils and polypeptide, for instance, contain amide groups that contribute to the bands at 1560 and 1650 cm^−1^. Diagnostic bands for lignin are those at 1660 cm^−1^ due to C=O stretching, and bands at 933 and 842 cm^−1^ can be attributed to -OH and -H (on aromatic ring) out-of-plane deformation, respectively [50].

**Figure 4:**
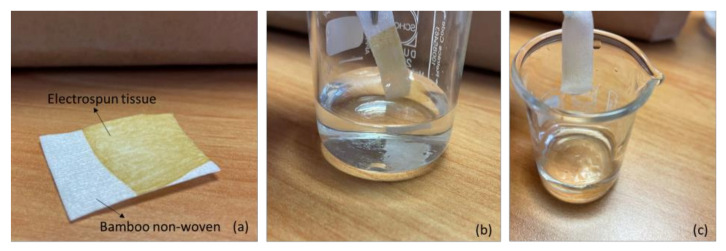
Trial performed with saline solution: (**a**) Structure of the sample; (**b**) strip before the immersion; (**c**) strip after 10 s.

**Figure 5:**
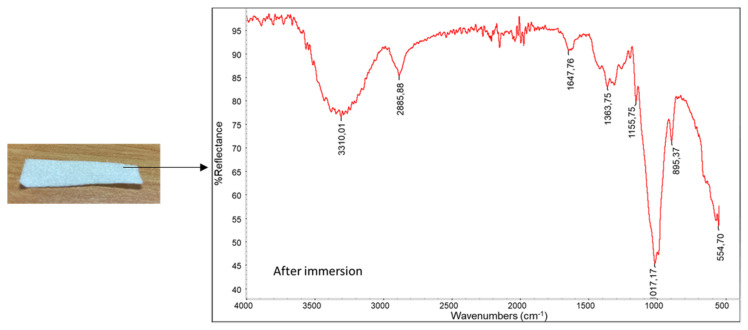
Infrared spectrum recorded onto the strip after immersion and drying in saline solution.

Strips of the bilayer system were immersed in distilled water, saline solution and in the liquid mimicking human saliva. After 10 s of immersion, the strips were removed, and they were white in color (Figure 4). The liquid remained almost transparent, indicating that the pullulan layer had the capacity to form a homogeneous solution/suspension in water. Then, the strips were dried and characterized by ATR spectroscopy (Figure 5).

The infrared spectrum result was very similar to that of the bamboo tissue, suggesting full release of the pullulan-based layer in the liquid. The tests demonstrated the capacity of the bilayer system for fast deliver of the active layer on a wet surface. Consequently, wet human skin (or any other body tissue) can rapidly receive pullulan and the active molecules from the bilayer system applied on it. Food grade pullulan is an additive named E1204.

**Figure 6:**
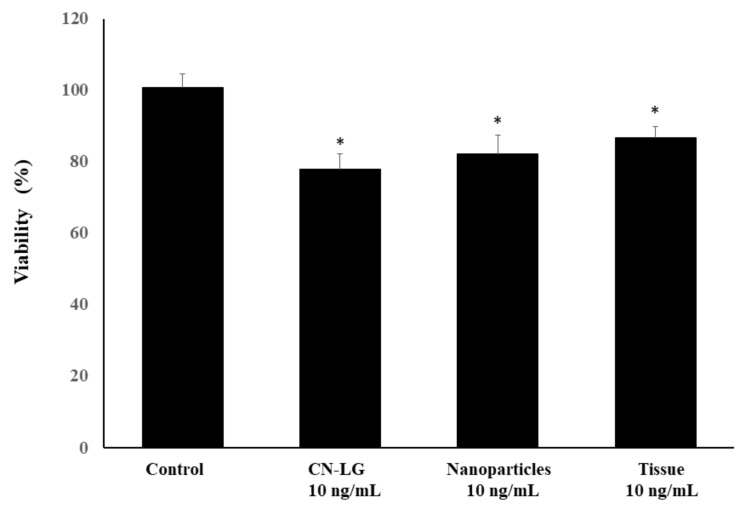
Effect of nanoparticles and bilayer tissue on the viability of human keratinocytes expressed as viability %, compared to the untreated control. Values having different symbols (anything, *, **, ***, etc…) belong to distinguishable samples. Significant vs. control (* *p* < 0.005).

Then, the bilayer system was characterized by performing viability tests using human keratinocytes, the most typical cells of the external skin layer (Figure 6). We investigated the effects of CN-LG, the nanoparticles loaded with allantoin, polypeptide and niacinamide as compared to the final tissue or the control. Viability slightly decreased with respect to the untreated control, with all the tested samples providing a similar result, since *p*-values were not significant among the CN-LG, nanoparticles and the final tissue.

**Figure 7:**
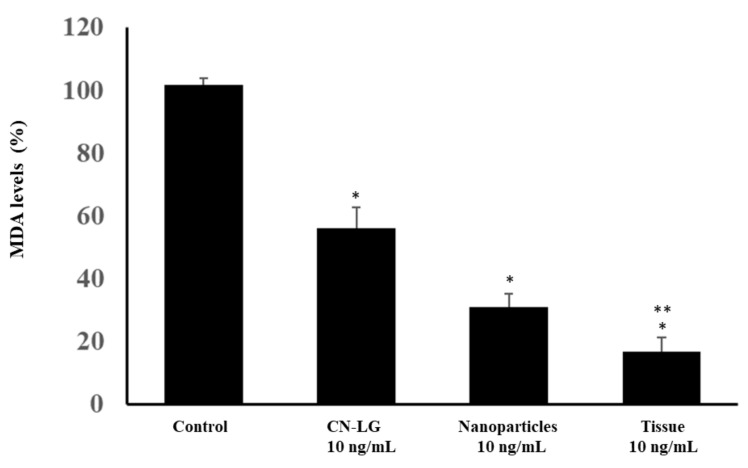
Protective antioxidant effects on peroxidation of linoleic acid in the presence of nanoparticles and the final tissue. Values having different symbols (anything, *, **, ***, etc…) belong to distinguishable samples. Significant vs. control (* *p* < 0.005); Significant vs. CN-LG and Nanoparticles (** *p* < 0.005). Increased antioxidant activities of CN-LG and CN-LG containing active molecules with respect to the control were found. Interestingly, the final tissue displays the highest activity in terms of antioxidant properties, suggesting that pullulan acts as a synergistic component. When the final tissue is compared to the CN-LG nanoparticles, the difference is significant, with *p* < 0.005. Regarding the comparison between CN-LG nanoparticles and the control, the difference is significant, with *p* < 0.0001.

The antioxidant activity of CN-LG nanoparticles, CN-LG-entrapping active molecules and the final tissue based on pullulan and bamboo fibers, were evaluated in vitro by using the total antioxidant potential (TRAP) technique. The principle of the method is based on the peroxidation of linoleic acid by the oxidant compound 2,2azobis-2,4-dimethylvaleronitrile (AMVN) with the formation of malondialdehyde (MDA) as the final product. Peroxidized linoleic acid was used as a control. MDA formation was detected, and the results are reported in Figure 7.

**Figure 8:**
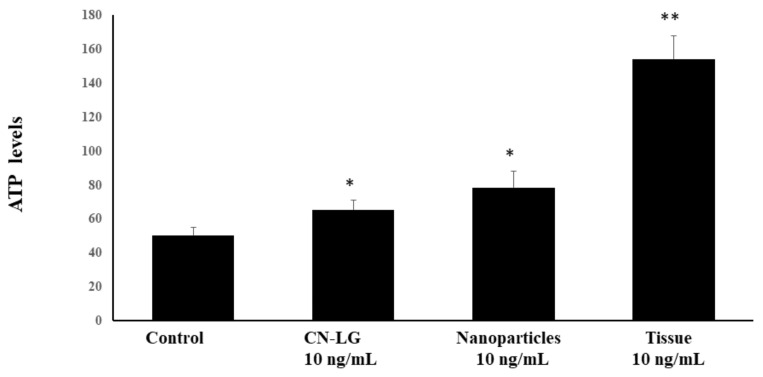
ATP levels in irradiated human keratinocyte cultures in the absence and the presence of pullulan tissue embedded with nanoparticles of CN-LG, encapsulating allantoin, nicotinamide and polypeptide from fish collagen. Results are reported as % of untreated control ATP content. Values having different symbols (anything, *, **, ***, etc…) belong to distinguishable samples. Significant vs. control (* *p* < 0.005); Significant vs. CN-LG and Nanoparticles (** *p* < 0.005).

The intracellular ATP content (Figure 8) was determined in irradiated keratinocytes in the absence and the presence of protective CN-LG complexes or the pure pullulan. Decreased ATP can be observed in the control keratinocytes exposed to irradiation, and ATP is significantly reduced in CN-LG and nanoparticles at a *p* < 0.05. This protection is significantly higher for the pullulan tissue treated with CN-LG incorporating active molecules (*p* < 0.0001) with respect to the untreated control, as compared to other treatments, showing a synergistic effect in this protective activity.

**Figure 9:**
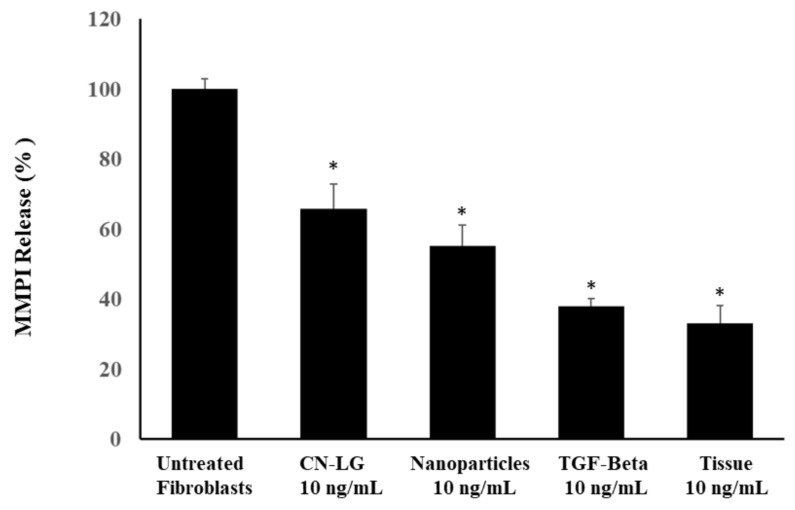
MMPI release of aged fibroblast treated with CN-LG carrier, nanoparticles and the final functional tissue. Values having different symbols (anything, *, **, ***, etc…) belong to distinguishable samples. Significant vs. control (* *p* < 0.005).

The release of metalloproteinase (MMPI) (Figure 9) is significantly decreased by the presence of CN-LG or all the other treatments (*p* < 0.005). The complex containing active molecules induces a significantly (*p* < 0.05) lower release than the CN-LG carrier. The final tissue shows a highly significant reduction (*p* < 0.005) in MMPI release compared to the complexes modified with active molecules. The difference between TGF-beta and the final tissue is not significant.

**Figure 10:**
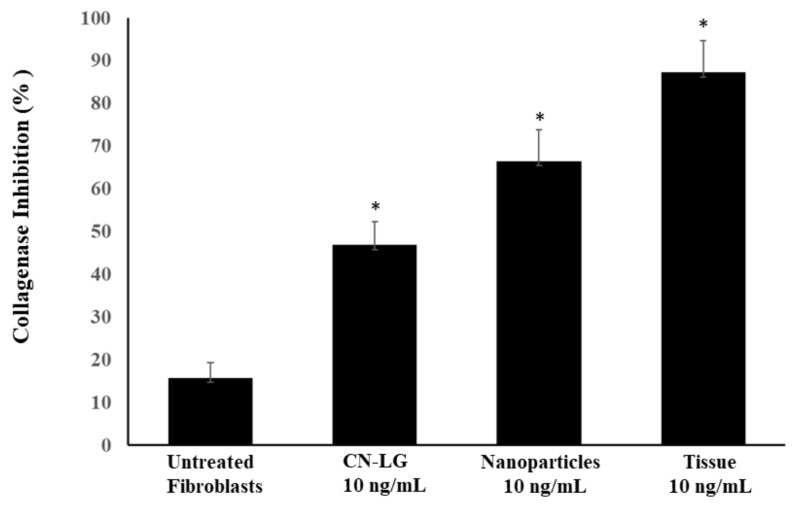
Percentage of inhibitory activity vs. collagen degradation in fibroblast cultures incubated with collagenase enzyme after treatment with CN-LG carrier, nanoparticles or the final tissue. Significant vs. control (* *p* < 0.005).

Inhibition of collagenase activity, tested on aged fibroblasts with our test compounds (Figure 10), shows higher inhibition with all treatments compared to the untreated control (*p* < 0.001). CN-Lg containing active molecules demonstrates significantly higher activity than the Cn-LG complex (*p* < 0.005), indicating that active molecules effectively contribute to collagenase inhibition. Even more so, the final tissue shows significantly higher (*p* < 0.005) inhibitory activity as compared to CN-LG containing active molecules. Thus, the presence of pullulan shows a synergistic effect on the inhibition of collagenase.

**Figure 11:**
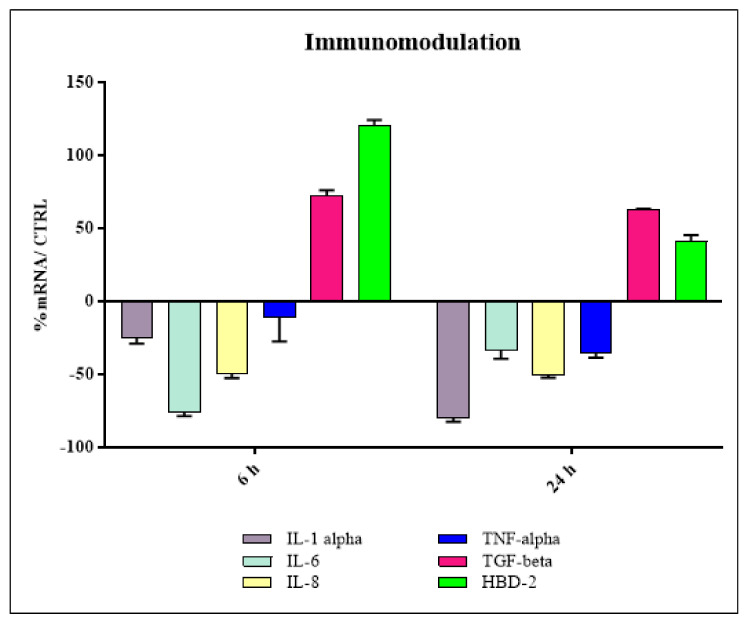
Levels of expression of the proinflammatory cytokines IL-8, IL-6, IL-1 α and TNF-α; anti-inflammatory cytokine TGF-β; and antimicrobial peptide HBD-2.

The immunomodulatory tests (reported in Figure 11, including their standard deviation bar) show that the fibers present strong anti-inflammatory activity, as they significantly downregulated all the proinflammatory cytokines tested and upregulated the anti-inflammatory cytokine TGF-beta. In addition, the fibers were able to strongly stimulate HBD-2 expression, suggesting the presence of indirect antimicrobial activity. In addition, the Alamar Blue assay shows that the fibers did not cause any type of damage to cell viability. In fact, the AB_RED_ value result was 100% for the control and 110% for the tissue sample.

## 4. Discussion

As shown from various studies, elastin and collagen synthesis results can be altered by photoaging and chronological aging, due to internal and external aggressions [51,52,53]. Consequently, general atrophy of ECM is generated with reduced synthesis of collagen/elastin and structural changes in their fibers, resulting in the visible appearance of fine lines and wrinkles [52]. ECM together with its fibers, in fact, gives the skin bulk, shape, strength and flexibility, and it is also the substrate for cell migration, differentiating, proliferation and survival by specific signals communicating with neighboring cells and the extracellular cytoskeleton [54,55,56,57,58]. Thus, collagen, due to its biological functions and capacity to regulate several signaling pathways, can be used together with its hydrolyzed peptides, to attempt to repair prematurely aged skin or skin affected by photoaging [54,55,56,57,58,59].

As a consequence, recovering natural and innovative active ingredients and carriers obtained from food-forestry wastes may be useful for treating and regenerating all aspects of the skin aging phenomena, without diminishing the Earth’s natural raw materials. Our proposed cosmeceutical tissues are aimed in this direction.

The active ingredients selected, in fact, are renewable, effective, safe and obtainable from waste materials. Moreover, by using these tissues as active carriers, it will be possible to greenify cosmeceuticals and nutraceuticals, reduce the footprints of existing carriers with renewable bio-based or recycled inputs, thus giving them more sustainable environmental, social and governance (ESG) values [60]. Consequently, fish peptides not only might be useful to increase the penetration of active ingredients, but also can probably act as signal molecules to stimulate the skin’s rejuvenation process. In fact, fish peptides may be able to carry specific innovative biological activities, allowing different types of molecules to selectively permeate the cell membrane, mimicking the effectiveness of natural peptides. Therefore, their mechanism of action might have the same functions that hormones and growth factors have to modulate the cellular selective processes for maintaining skin homeostasis [54,55,56,57,58,59].

In addition, nicotinamide might show various activities because, as a precursor of NAD, it may be able to prevent the depletion of cellular energy as a result of UV exposure [58], providing protection against UV-induced immunosuppression [61,62]. Nicotinamide might also play key roles in DNA repair and maintaining genomic stability by preventing chromatic structure. Moreover, nicotinamide-inhibiting sirtuins as NDA-dependent enzymes seem to play a critical role in cellular responses to environmental stressors modulated by various cytokines and by the expression of the tumor suppressor protein p53 [27,28,29,58,59,60,61,62,63]. Finally, as previously reported, allantoin has been reported to possess interesting antimicrobial, antioxidant and anti-inflammatory effectiveness, probably enhancing the same activities shown by the components of the carrier CN-LG embedded into pullulan tissue [28]. The obtained tissue, in fact, applied on wet skin and hydrolyzed by human enzymes, seems not only to act as a substrate for carrying the active ingredients and releasing them at the designed skin/mucous layer, but also provides the correct structural architecture that is useful for modulating the skin rejuvenation processes of prematurely aged or photoaged skin [64,65,66,67]. Embedding the CN-LG-loaded complexes in the PUL electrospun tissue seemed highly advantageous because a synergistic effect was observed in all the performed tests. This synergistic effect can be ascribed at the mechanism of release. If the solid nanostructured particles are suspended in the pullulan solution, they are certainly separated from each other due to the presence of the water-soluble pullulan in the filaments, since water will evaporate. Thus, during the application, when the particles are released, they are not agglomerated, and they show a very high surface to volume ratio. Thus, PUL behaves as an optimized carrier tissue for the loaded CN-LG complexes. We can consider that the active molecules (allantoin, fish collagen polypeptides and niacinamide) are doubly embedded in the nanostructured functional tissue, i.e., in the CN-LG complexes and in the PUL water-soluble carrier, both contributing to the mechanism of rapid and effective release, based on the high solubility of PUL, as well as the antioxidant, antimicrobial, anti-inflammatory and protective properties enhanced by the CN-LG carrier. The high solubility of PUL, similar to in Teno et al. work [28], and the nanostructured feature of the electrospun layer provide the possibility of commercializing this as a dry bilayer system in the form of a beauty mask or other releasing pads, so that preservatives and barrier fossil packaging, typical of product commercialized in wet conditions, are not necessary. Therefore, nanotechnology progress has given rise to the possibility of designing, producing and engineering innovative and different delivery systems for carrying various active ingredients. Naturally, the physicochemical properties of both carrier and ingredients have to be engineered at the molecular level, and their shape, size, superficial electrical charges and other parameters must be controlled to obtain the most effective and safest design of the final product [68]. However, due to the need to also respect the environmental ecosystem and to safeguard the Earth’s natural raw materials, utilization of waste material and reduced use of fossil-based plastics and the consequential increased production of greenhouse gas emissions have become a must for our society [69,70,71,72,73,74]. Thus, our proposal to use cosmeceutical tissues as new and smart carriers seems to be aimed in this direction, entering the category of the so-called hybrid cosmetics and beauty verse trends driven by the COVID-19 pandemic, the virtual economy and current financial principles [75,76].

## 5. Conclusions

An innovative bilayer system was proposed based on a bamboo nonwoven substrate. The investigated innovative cosmeceuticals can be realized using different natural polymers to make smart tissues. Thus, tissues loaded with various active ingredients, may be used indifferently in different fields such as medical devices, cosmeceuticals and nutraceuticals. The innovative tissue displayed antioxidant properties and compatibility with skin cells. Protective activity towards UV light, metalloproteinase release of aged fibroblasts and inhibitor activity against collagen degradation were also assessed. The immunomodulatory tests provided evidence of the anti-inflammatory activity of the bilayer system as well as its indirect antimicrobial activity. The effect of different concentrations of active biomolecules should be explored in the future in order to apply this innovative tissue in a specific sector. However, it is of fundamental importance to design not only different formulations based on the best biotechnological and physiological techniques and controlled by the right parameters, but also to respect the international rules governing the different products. Moreover, it is interesting to underline that these new vehicles are biodegradable; free of water, preservatives, emulsifiers, colors, fragrances and other chemicals; and can also be packed using paper or other organic materials. Additionally, the use of beauty devices has not to be forgotten, because from its worldwide growth of USD 51.3 billion in 2021, it is expected to reach USD 144.2 billion by 2028 with a compound annual growth rate (CAGR) of 18.8% [77,78]. In our opinion, these are the future trends and perspectives for a greener and sustainable planet.

## Figures and Tables

**Table 1:** Yield, loading, entrapment efficiency and mean size of the obtained nanoparticles embedding active molecules.

CN-LG Mean Size (nm)	Nanoparticles Yield (%)	Polypeptide Content (%)	Nicotinamide Content (%)	Allantoin Content (%)	Entrapment Efficacy (%)
185 ± 15	48 ± 8	18 ± 2	15 ± 3	14 ± 4	65 ± 6

All measurements were performed in triplicate; CN, chitin nanofibrils; LG, nano-lignin.

## Data Availability

Data are available from the corresponding author.
